# Food allergen protein families and their structural characteristics: new data from Europrevail

**DOI:** 10.1186/2045-7022-1-S1-S56

**Published:** 2011-08-12

**Authors:** Karin Hoffmann-Sommergruber

**Affiliations:** 1 Medical University of Vienna, Department of Pathophysiology, Vienna, Austria

## 

In the past decade the number of identified food allergens has tremendously increased. Nevertheless, these proteins can be assigned to only a limited number of protein families with certain biological functions such as hydrolysis of proteins/polysaccharides, binding, transport and storage of ligands and cytoskeleton association. In depth knowledge about the 3D structures of individual food allergens, their biological activity and stability will help to fine-tune in vitro diagnosis of food allergy and assess the risk of cross reactive allergies to other food sources. Therefore, the Europrevall project set out for a p collection of highly purified, characterised and authenticated food allergens from animal and plant food sources. The allergen library included both, recombinant and natural proteins. A catalogue of harmonised quality criteria of the purified allergens was agreed and up to date methodologies such as mass spectroscopy, circular dichroism spectroscopy, fourier-transform infrared spectroscopy and 1D-NMR analysis were applied to define them. Plant food allergens were purified from fruits (apple, peach, and kiwi), vegetables (carrot, celeriac, tomato and soybean) and grains, nuts and spices (wheat, hazelnut, peanut, walnut, sesame seeds, sunflower seeds and mustard). These allergens derived from the most important protein families such as the seed storage proteins from the prolamin and cupin superfamily, profilins, Bet v 1 related proteins, and oleosins. From animal foods allergenic proteins from cow’s and goat’s milk, hen’s egg, fish and shrimp were purified and included members of the following protein families: lipocalins, caseins, C-type lysozymes, various protease inhibitors, tropomyosins, and parvalbumins. Subsequently selected sets of allergens e.g .from kiwi or celeriac were used to compare conventional in vitro diagnosis with the allergen specific approach. Additionally, these well defined proteins were enabled further in depth studies investigating the impact of food processing or digestion on the structural features of the individual allergen.

